# Gynecologists’ attitudes toward and use of complementary and integrative medicine approaches: results of a national survey in Germany

**DOI:** 10.1007/s00404-020-05869-9

**Published:** 2020-11-17

**Authors:** Donata Grimm, Petra Voiss, Daniela Paepke, Johanna Dietmaier, Holger Cramer, Sherko Kümmel, Matthias W. Beckmann, Linn Woelber, Barbara Schmalfeldt, Ulrich Freitag, Matthias Kalder, Markus Wallwiener, Anna-Katharin Theuser, Carolin C. Hack

**Affiliations:** 1 Department of Gynecology and Gynecologic Oncology, Hamburg-Eppendorf University Medical Center, Martinistrasse 52, 20246 Hamburg, Germany; 2 Department of Gynecology and Obstetrics, Schleswig-Holstein University Medical Center, Campus Lübeck, Ratzeburger Allee 160, 23562 Lübeck, Germany; 3 Department of Internal and Integrative Medicine, Faculty of Medicine, Evangelische Kliniken Essen-Mitte, University of Duisburg-Essen, Am Deimelsberg 34a, 45276 Essen, Germany; 4 Department of Gynecology and Obstetrics, Klinikum Rechts Der Isar, Technical University of Munich, Ismaninger Strasse 22, Munich, Germany; 5 Breast Care Unit, Evangelische Huyssens-Stiftung, Kliniken Essen Mitte, Henricistrasse 92, 45136 MastologyEssen, Germany; 6 Department of Gynecology, Erlangen University Hospital, Friedrich Alexander University of Erlangen–Nuremberg, Comprehensive Cancer Center Erlangen-EMN, Universitätsstrasse 21–23, 91054 Erlangen, Germany; 7 Dipl. Med. Ulrich Freitag, State Association Chairman of the BVF, Private Practice, Turnerweg 11a, 23970 Wismar, Germany; 8 Department of Gynecology and Obstetrics, Phillips University of Marburg, Baldingerstrasse, 35033 Marburg, Germany; 9 Department of Obstetrics and Gynecology, University of Heidelberg, Im Neuenheimer Feld 672, 69120 Heidelberg, Germany; 10 Institute for Women’s Health (IFG) GmbH, Universitätsstrasse 21–23, 91054 Erlangen, Germany

**Keywords:** Integrative medicine, Complementary and alternative medicine, Gynecologic oncology, Breast cancer, Supportive care, Attitude

## Abstract

**Purpose:**

Despite patients’ widespread use and acceptance of complementary and integrative medicine (IM), few data are available regarding health-care professionals’ current implementation of it in clinical routine. A national survey was conducted to assess gynecologists’ attitudes to and implementation of complementary and integrative treatment approaches.

**Methods:**

The Working Group on Integrative Medicine of the German Society of Gynecological Oncology conducted an online survey in collaboration with the German Society of Gynecology and Obstetrics (DGGG) in July 2019. A 29-item survey was sent to all DGGG members by email.

**Results:**

Questionnaires from 180 gynecologists were analyzed, of whom 61 were working office-based in private practice and 95 were employed in hospitals. Seventy percent stated that IM concepts are implemented in their routine clinical work. Most physicians reported using IM methods in gynecological oncology. The main indications for IM therapies were fatigue (*n* = 98), nausea and vomiting (*n* = 89), climacteric symptoms (*n* = 87), and sleep disturbances (*n* = 86). The most commonly recommended methods were exercise therapy (*n* = 86), mistletoe therapy (*n* = 78), and phytotherapy (*n* = 74). Gynecologists offering IM were more often female (*P* = 0.001), more often had qualifications in anthroposophic medicine (*P* = 0.005) or naturopathy (*P* = 0.019), and were more often based in large cities (*P* = 0.016).

**Conclusions:**

There is strong interest in IM among gynecologists. The availability of evidence-based training in IM is increasing. Integrative therapy approaches are being implemented in clinical routine more and more, and integrative counseling services are present all over Germany. Efforts should focus on extending evidence-based knowledge of IM in both gynecology and gynecological oncology.

**Electronic supplementary material:**

The online version of this article (10.1007/s00404-020-05869-9) contains supplementary material, which is available to authorized users.

## Introduction

The overall use of complementary and alternative medicine (CAM) has increased noticeably worldwide in recent years, and evidence on its effectiveness has started to be incorporated into medical treatment guidelines [1–6]. The recommendations on the treatment of breast cancer published by the Breast Committee of the Working Group on Gynecological Oncology (*Arbeitsgemeinschaft Gynäkologische Onkologie,* AGO) already included complementary methods in 2002. In addition, the German Cancer Society (*Deutsche Krebsgesellschaft*, DKG) is developing a guideline for IM that includes various medical professions [7].

*Complementary* medicine refers to health-care practices that traditionally have not been part of conventional medicine and represent forms of treatment that are used together with *conventional* medicine. In contrast, *alternative* medicine refers to non-mainstream practices that are generally not considered standard medical approaches and are used instead of *conventional* medicine [8–10]. According to the National Center for Complementary and Integrative Health (NCCIH), integrative medicine (IM) differs from CAM because it combines conventional and complementary treatments in a coordinated way [11]. Neither rejecting conventional therapies nor relying on alternative medicine, IM adopts only those complementary modalities that are supported by the strongest evidence of safety and effectiveness, resulting in a supplementary, holistic approach to oncological treatment [1, 12–14]. We therefore prefer to use the term IM instead of CAM.

The Working Group on Integrative Medicine (*Arbeitsgemeinschaft Integrative Medizin,* AG-IMed) of the German Society for Gynecology and Obstetrics (*Deutsche Gesellschaft für Gynäkologie und Geburtshilfe,* DGGG) identifies CAM treatment on the basis of published evidence-based research that is suitable for supplementing the portfolio of conventional medicine [1].

Physicians represent the main providers of IM therapy in Germany [1, 15] and, in contrast to other countries, breast cancer in Germany is treated by onco-gynecologists. Naturopathy, acupuncture, nutritional counseling, homeopathy, and manual therapy/chiropractic are recognized qualifications for physicians in Germany. While naturopathy is used for different and often eclectic treatment approaches internationally [16], it mainly encompasses herbal medicine (also called phytotherapy), hydrotherapy, and mind–body medicine counseling in Germany [17]. While not a recognized qualification for physicians, anthroposophic medicine is commonly used by German physicians. This medical system is based on a specific organismic concept and uses drugs derived from herbal, mineral, and animal sources, eurythmy (a specific movement therapy), art therapy, rhythmical massage, and lifestyle recommendations [18] (Supplementary digital file 2). No formal qualification is mandatory to practice IM in Germany.

Previous studies on IM use have mainly focused on oncological patients’ motivation, objectives, information sources, and characteristics [19–24]. However, little is known about the acceptance and use of IM by gynecological oncologists and general gynecologists throughout Germany [19, 25]. While patients often request IM, many physicians and other caregivers are hesitant to apply any IM methods, especially in a curative setting. A study conducted by Furlow et al., surveyed 401 obstetrics/gynecology physicians in the state of Michigan. Physicians appeared to have a positive attitude toward IM and the majority indicated that they had referred patients for at least one IM modality. Around 73.2% of physicians stated that IM includes areas and methods from which conventional medicine could benefit [26]. The modalities that were most commonly regarded as being highly or moderately effective were biofeedback, chiropractic, acupuncture, meditation, and hypnosis/guided imagery. Physicians (83%) indicated that they routinely ask their patients about IM use [26]. However, the majority of patients did not consult their health-care provider before initiating an IM method elsewhere. The reason for this given by patients was that their physicians never asked them about the use of IM [26]. There is an obvious discrepancy, possibly due to physicians’ time constraints and/or lack of reimbursement for IM. A recent study by Hack et al. confirmed that aspects of IM still very rarely form part of oncological consultations, and this in turn discourages patients. IM programs in comprehensive cancer centers might solve such problems [27].

The aim of this cross-sectional study was to evaluate the current state of attitudes toward IM and patterns of IM provision by office-based as well as hospital-based physicians throughout Germany. Further characteristics such as age, gender, duration of professional work, and experience of physicians perceiving IM as effective were also analyzed. In addition, indications for IM use, competences, structures, implementation, and qualifications, as well as the patients’ expectations (as perceived by the gynecologists) were assessed. Finally, the financial reimbursement provided for IM therapies in daily routine was analyzed. The data collected represent the current situation in the provision of IM by gynecologists in Germany.

## Materials and methods

The Working Group on Integrative Medicine of the German Society of Gynecological Oncology (IMed) conducted an online survey in collaboration with the DGGG. The IMed Committee was founded on June 28, 2013. This group of gynecological oncologists focuses on the clinical, scientific, and organizational aspects of IM in oncology. It supports scientific research and cooperation in the field of IM and also encourages the implementation of evidence-based integrative therapy approaches and regular IM consultation hours, to integrate these into standard oncological care [19].

In July 2019, a self-administered, 29-item online survey was sent to all members of the DGGG. The email was sent on July 17th, and a reminder email was not sent. Participation was voluntary and anonymous.

**Fig. 1:**
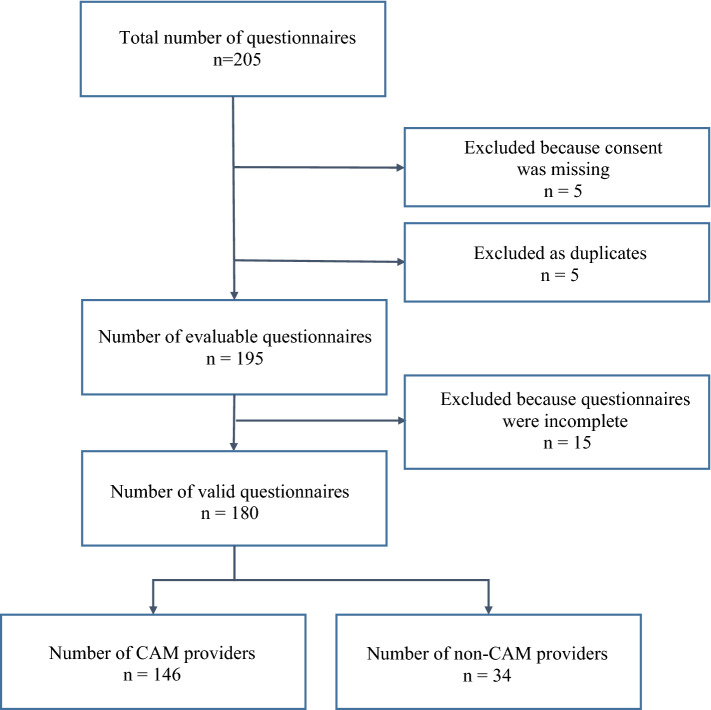
Flow chart

The survey contained 15 multiple-choice questions, including items on the use of integrative therapy methods, fields of indications, counseling services, level of specific qualifications, etc., as well as 14 sociodemographic questions. Questions were designed with a multiple-choice entry format, with single or multiple answers. Missing values were allowed. However, in cases of suspected duplication or when values were missing in all questionnaire items, these questionnaires were deemed unsatisfactory and excluded (Fig. 1). The online platform “SoSci Survey” ensured data transmission at any time in accordance with the current state of technology, and study participants were determined using unique visitors by generating a code with regard to name and date of birth at the start of the survey. The time needed to complete the survey was approximately 12 min. Explanations of terms with regard to the topic of integrative and complementary medicine can be found in Supplementary digital file 2.

The study protocol was in accordance with the Declaration of Helsinki, and ethical approval was obtained from the Hamburg Medical Association (reference number Hamburg PV5847). Informed consent was obtained from each participant prior to participation in the study.

### Statistical analysis

Statistical evaluation consisted of descriptive analysis. Total amounts and percentages were calculated. Patients with missing values were excluded from the analysis of the corresponding variables. Student’s independent *t *tests were used to assess differences in age and years of professional experience between physicians who were providing and not providing IM methods. Differences in categorical variables between physicians providing and not providing IM methods were tested using chi-squared tests or Fisher’s exact test, as appropriate. The level of significance was set at < 0.05. Data were analyzed using IBM SPSS Statistics, version 24.0.0.2 (IBM Corporation, Armonk, New York, USA).

## Results

A total of 205 questionnaires were returned, 25 of which were excluded from the evaluation either because of suspected duplication or because values were missing in all questionnaire items. Details on evaluable questionnaires are provided in the flow chart (Fig. 1). In all, 180 gynecologists completed the survey, with 102 women (57%) and 48 men (27%). Thirty participants (17%) gave no details on their gender. The respondents’ age ranged from 20 to 74 years (median 43 years). A total of 101/180 gynecologists (56%) had at least a doctoral degree (Table 1).

**Table 1:** Sociodemographic and professional characteristics of physicians providing or not providing integrative medicine (IM) (*n* = 180)

Variable	Providers of IM treatments	Not providers of IM treatments	*P*
Participants (*n*, %)	146 (81%)	34 (19%)	
Age (mean, SD)	44.9 (11.9)	41.1 (10.4)	0.114
Age (median, range)	44 (20–74)	37 (28–67)	
Gender (*n*, %)			
Male	25 (17%)	23 (67%)	
Female	95 (65%)	7 (21%)	
Missing	26 (18%)	4 (12%)	
Academic qualifications (*n*, %)			
Doctoral degree	70 (48%)	18 (53%)	
Adjunct professor	3 (2%)	2 (6%)	
Professor	6 (4%)	2 (6%)	
No doctoral degree**	39 (27%)	8 (23%)	
Missing	28 (19%)	4 (12%)	
Educational status (*n*, %)			
Resident	26 (18%)	10 (29%)	
Specialist	89 (61%)	20 (59%)	
Missing	31 (21%)	4 (12%)	
Additional qualifications (*n*)*			
Acupuncture	21	2	
Anthroposophic medicine	26	0	
Emergency medical aid	1	0	
Homeopathy	7	0	
Naturopathic treatments	28	1	
Nutritional medicine	6	4	
Psycho-oncology, psychosomatic medicine, psychotherapy	15	4	
Other	27	5	
Years of professional work (mean, SD)	15.3 (10.2)	13.2 (9.9)	
Years of professional work (median, range)	14 (0–40)	10 (1–36)	
Workplace (*n*) *			
Office-based	54	7	
Hospital-based	73	22	
Missing	19	5	
Certified breast cancer center (*n*, %)			
Yes	60 (41%)	17 (50%)	
No	51 (35%)	13 (38%)	
No information	3 (2%)	0 (0%)	
Missing	32 (22%)	4 (12%)	
Certified gynecological-oncology center (*n*, %)			
Yes	49 (34%)	12 (35%)	
No	55 (38%)	16 (47%)	
No information	5 (3%)	1 (3%)	
Missing	37 (25%)	5 (15%)	
Location of the office (*n*, %)			
Large city (population > 100,000)	55 (38%)	23 (67%)	
Medium-sized City (population 20,000–100,000)	36 (25%)	4 (12%)	
Town (population 5000–20,000)	24 (16%)	1 (3%)	
Rural region (population < 5000)	2 (1%)	1 (3%)	
Missing	29 (20%)	5 (15%)	

*Multiple responses possible, therefore no percentages are reported

**In Germany, an MD is not automatically awarded upon completion of medical school. As this degree is awarded only after an additional exam has been passed, it is possible to practice as a physician without having an MD

Among the participants surveyed, 146 (81%) stated that they provided IM approaches in clinical practice. Gynecologists offering IM were more often female, more often had qualifications in anthroposophic medicine or naturopathy, and were more often based in large cities than those who did not offer IM (Table 1). Sixty-five percent of the female gynecologists surveyed provided IM, in comparison with 17% of the male gynecologists surveyed (Table 1).

Sixty-one out of 180 gynecologists worked in private office practices and 95 were employed in hospitals. Three participants stated that they worked both in an office practice and a hospital, and nine participants stated they were neither office-based nor hospital-based; 77/180 (43%) worked in certified breast cancers centers and 61/180 (34%) in certified gynecological oncology centers (Table 1). Most of the gynecologists surveyed were already specialists and had been working for a mean of 14.9 years.

**Fig. 2:**
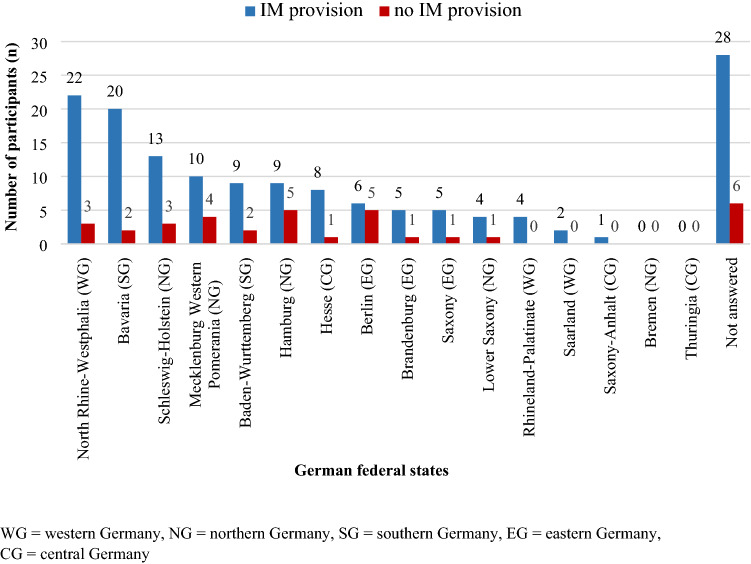
Distribution of gynecologists surveyed in Germany who provide integrative medicine (IM; *n* = 146, 81%) or do not provide IM (*n* = 34, 19%). *WG* western Germany, *NG* northern Germany, *SG* southern Germany, *EG* eastern Germany, *CG* central Germany

With regard to regional differences, it was found that most participants surveyed were from the state of North Rhine–Westphalia (*n* = 22, 12%) in western Germany; Bavaria (*n* = 20, 11%) in southern Germany; and Schleswig–Holstein (*n* = 13, 7%) in northern Germany (Fig. 2).

Seventy percent of the gynecologists surveyed (*n* = 94) providing IM (*n* = 134, 12 missing) stated that they had implemented IM in their routine clinical work, versus 25% (*n* = 34) who did not include it in the clinical routine and 5% (*n* = 6) who did not provide any information on the issue. Participants who had established routine IM procedures had been offering these procedures for an average of 10.9 ± 8.7 years. Ninety-five gynecologists (71%) stated that they routinely informed their patients about IM treatment approaches at any time during diagnosis and treatment, whereas 30/134 (22%) started counseling only if patients asked for it, and three participants (2%) stated that they only discussed IM options if conventional medical methods had been insufficient or had failed. Only 5% (*n* = 6) did not provide any further information (data not shown in a table).

Counseling on applicable IM therapies was mainly provided by the gynecologists themselves (*n* = 130); by collaborating partners such as other hospitals, clinics, or nonmedical practitioners (*n* = 43); or by breast care nurses (*n* = 41) (Table 2). The additional qualifications most often held by those providing IM counseling were naturopathy (*n* = 63), acupuncture (*n* = 46), and anthroposophic medicine (*n* = 39) (Table 2).

**Table 2:** Characteristics of professionals providing counseling on integrative medicine (IM) therapy (*n* = 146)

	*n*
Professions frequently providing counseling on IM therapies*	
Breast care nurse**	41
Diet assistant**	14
Gynecologist	130
Mind–body therapist**	12
Nutritionist**	23
Other	24
Referral to cooperating partner (e.g., alternative practitioner, other hospital, etc.)	43
Sports scientist**	9
Study nurse**	19
Additional qualifications of professionals providing advice on IM therapies*	
Acupuncture	46
Anthroposophic medicine**	39
Homeopathy**	32
Manual therapy/chiropractic	11
Naturopathic therapy**	63
Neural therapy	16
Nutritional counseling	29
Other	20
Phytotherapy**	32
Traditional Chinese medicine	16

Only participants who answered “ Yes” to the question “Do you offer complementary medical treatment methods in your hospital or practice?” are included in the analysis (*n* = 146)

*Multiple responses possible, therefore no percentages are reported

**For explanations, see supplementary digital file 2 (S2)

Most providers of IM therapy treatments 83/134 (62%) estimated that counseling was not cost-effective, 15% (*n* = 20) considered that IM counseling would be cost-neutral, and 23% (*n* = 31) did not supply this information (data not shown in a table).

Participants were also asked to rate the fields in which, and for which problems, IM was a reasonable treatment option in gynecology and gynecological oncology. The results are presented in Table 3. For gynecological complaints such as climacteric symptoms (*n* = 102), premenstrual syndrome (*n* = 80), hormonal dysregulation (*n* = 79), and urinary tract infection (*n* = 75), IM therapies are most commonly regarded as a reasonable option. By contrast, polycystic ovary syndrome (*n* = 43), infertility (*n* = 42), and incontinence (*n* = 38) were thought by most participants to have less relevance as possible indications for IM (Table 3).

**Table 3:** Recommendations for various integrative medicine (IM) methods in relation to gynecological (*n* = 133^a^) and gynecological-oncology (*n* = 110^b^) indications

	*n*
In general gynecology*^a^	
Climacteric symptoms	102
Premenstrual syndrome	80
Hormonal dysregulation	79
Urinary tract infection	75
Genital infection	54
Endometriosis	55
Polycystic ovary syndrome	43
Infertility	42
Incontinence	38
No, I do not use IM therapies for the above indications	16
Other	10
In gynecological oncology *^b^	
Fatigue	98
Nausea and vomiting	89
Climacteric complaints	87
Sleeping disorders	86
Psychological complaints—e.g., anxiety, depression	82
Loss of appetite	83
Polyneuropathy	79
Joint pain	73
Abdominal discomfort (constipation/diarrhea and pain)	63
Cognitive impairments	62
Mucositis	58
(Tumor) pain	54
Skin changes (e.g., radiation-induced dermatitis)	51
Hand–foot syndrome	40

^*^Multiple responses possible, therefore no percentages are reported

^a^Only participants who answered “Yes” to the question “Do you offer complementary medical treatment methods in the field of general gynecology?” are included in the analysis (*n* = 133). Missing values were not taken into account

^b^Only participants who answered “Yes” to the question “Do you use complementary medical treatment methods in the field of gynecological oncology?” were included in the analysis (*n* = 110). Missing values were not taken into account

Most of the gynecologists surveyed (*n* = 113) reported that they used IM therapy methods in the field of gynecological oncology in patients with breast cancer (*n* = 112), ovarian cancer (*n* = 93), cervical cancer (*n* = 62), endometrial cancer (*n* = 79), peritoneal and Fallopian tube cancer (*n* = 72), and vulvar/vaginal carcinoma (*n* = 66) (data not shown in a table). The main indications for IM therapies were fatigue (*n* = 98), nausea and vomiting (*n* = 89), climacteric symptoms (*n* = 87), sleep disturbances (*n* = 68), and psychological complaints such as anxiety and depression (Table 3). Complementary, the Supplementary digital file 3 displays integrative therapies used during and after breast cancer treatment, with levels of evidence on complementary medicine while breast cancer is the area in which most research of this kind has been carried out.

The most commonly recommended methods in the field of general gynecology, such as phytotherapy (*n* = 81), exercise therapy (*n* = 76), and food supplements (*n* = 66), are listed in Table 4. The most commonly recommended IM methods in the field of gynecologic oncology were exercise therapy (*n* = 86), mistletoe therapy (*n* = 78), and phytotherapy (*n* = 74) (Table 4).

**Table 4:** Recommendations for integrative medicine (IM) treatment in the fields of general gynecology (*n* = 113*^a^) and gynecologic oncology (*n* = 110*^b^)

	*n*
Recommended methods in general gynecology*^a^	
Biological therapies	
Phytotherapy	81
Supplements (vitamins, minerals, trace elements, amino acids, fatty acids)	66
Mistletoe therapy	45
Cancer diet	9
Manipulative and body-based therapies	
Sports/exercise therapy	76
Manual therapies (massage/lymph therapy)	51
Osteopathy/chiropractic/craniosacral therapy	45
Neural therapy	24
Mind–body intervention	
Relaxation processes—e.g., progressive muscle relaxation	60
Yoga	51
Autogenic training	41
Meditation	38
Creative therapy (art/music)	30
Qigong, tai chi	24
Biofeedback	23
Hypnosis	13
Reiki	7
Medical systems^c^	
Traditional Chinese medicine (including acupuncture/acupressure)	52
Anthroposophic medicine	48
Classic homeopathy	41
Kneipp therapies	28
Ayurveda	9
Other methods	
Wraps/pads	44
Aromatherapy	32
Autologous blood	14
Hyperthermia	12
Other	6
Recommended methods in gynecologic oncology*^b^	
Biological therapies	
Mistletoe therapy	78
Phytotherapy	74
Supplements (vitamins, minerals, trace elements, amino acids, fatty acids)	66
Cancer diet	13
Manipulative and body-based therapies	
Sports/exercise therapy	86
Manual therapies (massage/lymph therapy)	67
Osteopathy/chiropractic/craniosacral therapy	39
Neural therapy	24
Mind–body intervention	
Relaxation processes—e.g., progressive muscle relaxation	59
Meditation	54
Yoga	52
Autogenic training	46
Creative therapy (art/music)	38
Qigong, tai chi	34
Biofeedback	21
Hypnosis	14
Reiki	9
Medical systems^c^	
Traditional Chinese medicine (including acupuncture/acupressure)	52
Anthroposophic medicine	51
Classic homeopathy	39
Kneipp therapies	32
Ayurveda	8
Other methods	
Wraps/pads	52
Aromatherapy	38
Hyperthermia	20
Autologous blood	10
Others	5

^*^Multiple responses possible, therefore no percentages are reported

^a^Only participants who answered “Yes” to the question “Do you offer complementary medical treatment methods in your hospital or practice?” were included in the analysis (*n* = 113). Missing values were not taken into account

^b^Only participants who answered “Yes” to the question “Do you use complementary medical treatment methods in the field of gynecological oncology?” were included in the analysis (*n* = 110). Missing values were not taken into account

^c^Medical systems are explained in more detail in Supplementary digital file 2 (S2)

Most physicians used IM during chemotherapy (*n* = 100), hormone therapy (*n* = 98), and aftercare/follow-up (*n* = 92) (Supplementary digital file 4).

Last but not least, the gynecologists were asked about their patients’ expectations of IM. IM providers reported their patients’ leading expectations to be an improvement in the quality of life (*n* = 119), followed by the wish to have holistic treatment (*n* = 98) and the strengthening of their immune system (*n* = 96) (Table 5).

**Table 5:** Patients’ expectations of the use of integrative therapies* (as reported by their gynecologists providing IM treatments for their patients)

	*n*
Improved quality of life	119
Intention to have holistic treatment	98
Strengthening of the immune system	96
Improved stress and disease management	88
Desire to do something for themselves	87
Promotion of cancer healing	73
Healing with self-help	65
Dissatisfaction with standard therapy methods	55
Alleviation of side effects of the cancer therapy	49
Prolongation of life	2

The analysis only included responses in which the answer to the question “Do you provide complementary medical treatment methods in your hospital/office?” was “Yes” (*n* = 135). Therefore those gynecologists not providing IM could not be considered. Missing values were not taken into account

^*^Multiple responses possible, therefore no percentages are reported

## Discussion

This national survey represents an attempt to describe attitudes toward IM and patterns of IM provision by office-based as well as hospital-based gynecologists and gynecological oncologists, most likely the main providers of IM for gynecological cancer patients. Little has so far been known about current gynecological providers’ characteristics, as well as their attitudes and user behavior in Germany. So far, professional integrative counseling and therapy concepts have rarely been available in hospitals in Germany and are often limited to a few selected breast cancer centers and specialized hospitals for IM [28]. As it has now been shown by several published studies that gynecological patients are in favor of IM, the evaluation of IM supply by health professionals in Germany is a matter of major interest.

The Working Group on Integrative Medicine of the German Society of Gynecological Oncology earlier developed a questionnaire for gynecological oncologists to evaluate the degree of acceptance, usage, and implementation of IM. The questionnaire was successfully distributed in 2014 to all members of the German Society of Gynecological Oncology in the German Cancer Society (DKG), with a focus on gynecological oncologists working in hospitals [7, 19]. The study showed that there is considerable interest in IM among gynecological oncologists, but that IM therapy approaches were poorly implemented in routine clinical work (25%). However, 64.7% of the gynecological oncologists were planning to do so [19]. In addition, although not routinely, 93% reported that they use IM therapy methods with breast cancer patients and 80% that they use them with ovarian cancer patients. IM providers tended to be male (67.3%) rather than female (32.7%), and 76% were working in certified breast cancer centers.

When the present data are compared with the data from the 2015 survey—as a follow-up study after 5 years—it is evident that 81% of the participants surveyed were providers (vs. non-providers) of IM; 70% were implementing IM in their routine clinical work, and tended to be female rather than male (65% vs. 17%). They were also slightly older than in the earlier AGO survey. Most were working in hospitals rather than being office-based, were in certified breast cancer centers in the majority of cases (41% vs. 35%), and were living in larger cities rather than smaller ones (63% vs. 17%). However, only gender and location differed significantly between providers and non-providers.

A similar survey conducted in 1998–1999 by Münstedt et al., including physicians in various medical fields (including gynecologists, both hospital and office-based) found significant differences between IM providers and non-providers with respect to gender (male 56% vs. female 48.3%), age (providers were older than non-providers), and place of work (office-based 73.4%, vs. hospitals 43.2%, vs. university hospitals 34.7%) [15]. In comparison with the previous studies, the present results show that in the past 20 years, IM has been increasingly integrated into cancer care, particularly in hospitals. However, the studies are not fully comparable, as gynecologists were only a subgroup in the earlier analysis.

A study by Huber et al. surveyed the attitudes of young general practitioners in Germany toward IM [29]. The data indicated that experienced older general practitioners had made a shift from primarily disease-centered to more person-centered care [29, 30].

Recent data from 2014 on IM in radiotherapy in Germany showed that for 32.2% of gynecological oncologists, IM is part of routine treatment (not part of it, 57.3%; unknown, 10.5%) and that 22.0% were planning to incorporate it [7]. Like the Society of Gynecology and Obstetrics, the German Society of Radio-Oncology and Radiotherapy (*Deutsche Gesellschaft für Radioonkologie,* DEGRO) has recently set up a guideline commission for IM, and radiation oncology is a key field in this context [7].

Relative to the different federal states in Germany, it appears that North Rhine–Westphalia, Bavaria, Schleswig–Holstein, Baden-Wurttemberg, and Hamburg are playing a pioneering role in providing integrative counseling in an oncologic setting (Fig. 2). This is not surprising, as one of the best-established naturopathic hospitals in Germany is located in Essen in North Rhine–Westphalia (“Integrative Onkologie KEM/Evangelische Kliniken Essen-Mitte”). Moreover, the south of Germany has historically shown greater interest in naturopathic treatments than the north. However, due to the high demand from patients and increasing training opportunities for gynecologists, IM has now also reached the north. In recent years, many (university) medical centers in the north such as Schleswig–Holstein and Hamburg-Eppendorf, for example, have established qualified counseling units for IM in gynecology—following the south of Germany, where such units have already existed for more than 10 years (e.g., the university medical centers in Erlangen and at the Technical University of Munich). However, it should be borne in mind that North Rhine–Westphalia and Bavaria have the largest populations among Germany’s federal states, and this may have skewed the data.

At best, gynecologists with appropriate training (e.g., by the Working Group on Gynecological Oncology/AGO) and with qualifications can offer integrative oncology care. Less optimally, physicians without evidence-based knowledge may provide counseling on IM therapies.

In contrast to the special curricula that apply in naturopathy or nutrition, for example, with an examination by the regional state medical association, comparable standardized quality and qualifications for IM counseling in oncology, general gynecology, or obstetrics do not exist. However, the Working Group on Integrative Medicine of the German Society of Gynecological Oncology has recently established a certified course in “Integrative Medicine in Oncology” to correct the current shortage and train medical staff in integrative oncology, to enable them to implement certified IM counseling units both hospital-based and office-based settings to meet the high demand and necessary quality for integrative counseling in Germany. In the best cases, additional qualifications are available.

In addition to the shortage of services and qualified medical staff, another potential hazard that is well known in association with integrative counseling is communication difficulties between physicians and patients [19]. Recent data showed that 47–85% of women with breast cancer who used IM did not disclose this use to the doctors treating them [31]. In the field of general gynecology, only 51.8% of women disclosed their use of IM [32, 33]. Moreover, physicians have in the past had little interest in initiating communication about unconventional therapies, with most regarding such a discussion as a poor use of their time [34]. Although patients want counseling on IM therapies from their gynecologists, rather than from friends, media, self-help groups, etc., gynecologists and other physicians complain about a lack of time, concepts, experience, and last but not least a lack of adequate remuneration [1, 19]. Similarly, in this cross-sectional study, most providers of IM (63%) estimated that counseling would be not cost-effective (AGO survey 55%, DEGRO survey 37.8%), and only 15% considered IM counseling to be cost-neutral (DEGRO 9.8%). Future studies could compare the perceived percentage of use and the disclosure of use by physicians and real percentages from patient surveys.

Among the gynecologists providing IM, 71% stated that they informed their patients about IM at various times during diagnosis and treatment, whereas 22% only started counseling if the patients asked about it proactively. Two percent of the participants, however, only started such a conversation if conventional medical methods were insufficient or had failed (data not shown in a table). Although at a far lower level, this is in line with the results reported by Kalder et al. that 40% (vs. 2% today) of gynecologists recommended IM because of the ineffectiveness of conventional therapies, as an expression of helplessness when the limits of conventional treatment options had been reached [1]. However, the motivation for IM should never be desperation, since helpful palliative medical options have been developed in the field of oncology in particular [35].

IM use and counseling in oncology in the present study were mainly present during chemotherapy (AGO 84%, DEGRO 80%), hormone therapy (AGO 60%, DEGRO 46.2%), and aftercare/follow-up (AGO 70%, DEGRO 55.2%), but continued through all phases of treatment (Supplementary digital file 4)—underlining the strong demand from patients in all treatment phases. The reasons for using IM concomitantly with conventional treatment or after primary therapy are mostly not for medical treatment of the disease, but rather as a supportive treatment to eliminate symptoms, reduce side effects, and strengthen the immune system [36]. Other reasons given by patients for using IM include physical and psychological support for the body and general well-being, improving quality of life, relieving chemotherapy-induced symptoms, enhancing the immune system, and even increasing the chances of survival [37, 38].

This again emphasizes, on the one hand, the need for implementation of qualified and certified IM counseling units, and on the other hand the need for interdisciplinary collaboration among physiotherapists, nutritionists, psychologists, office-based gynecologists, general practitioners, and so on. It is surprising that IM counseling services exist without adequate interdisciplinary collaboration to combine services for patients who request them.

Implementation of office-based units is important, since long-term relationships between most gynecologists and patients may offer a deeper basis of trust to enable patients to ask about integrative methods without inhibitions. Regardless of the cancer type, treatment phase, or workplace, IM therapy methods need to be implemented more in official treatment guidelines to promote trust around physicians as well as patients. At the same time, education and training are mandatory to enable physicians to implement these evidence-based IM methods [39]. In addition, IM treatment approaches need to be implemented in routine clinical work to promote adherence to proposed therapies that meet patients’ urgent needs. Remuneration for IM therapies also needs to be discussed by health-care insurance bodies.

The view that there is an urgent need to provide better qualifications and training for providers of IM in gynecology is not new [25]. The increasing numbers of qualifications available in IM appear to be a positive response to this. The main additional qualifications observed in the present cohort were in naturopathic therapy *n* = 63 (AGO survey 2014, 48.6%), followed by acupuncture at *n* = 46 (AGO survey 2014, 29.2%) and anthroposophic medicine at *n* = 39 (AGO survey 2014, 13.9%) (Table 2). These figures are higher than those in a similar survey by Münstedt et al., which was conducted six years earlier but in a different cohort (310 gynecologists and obstetricians from the state of Hesse in Germany). In that study, 14.8%, 6.1%, and 2.6% had received qualifications in acupuncture, naturopathy, and homeopathy, respectively.

To date in 2020, most research concerned the group of breast cancer patients. The most relevant practical skills seem to be exercise therapy, yoga, nutritional counseling, mindfulness-based stress reduction, acupuncture as well as hypnosis. According to existing Level of Evidence A, the recommendation of these skills for physicians might be meaningful (Supplementary digital file 3) [40–42]. Besides international guidelines—in Germany for example—the Breast Committee of the Working Group on Gynecological Oncology (AGO) annually updates evidence-based recommendations on complementary therapies (an English version is available as well) [41, 42]. This is in addition to international guidelines. It would therefore be advisable that physicians obtain qualifications in these IM approaches or collaborate with certified providers.

Data on IM are scarce in the field of gynecology. The participants in the present survey mentioned climacteric symptoms, premenstrual syndrome, hormonal dysregulation, urinary tract infections, genital infections, and endometriosis as reasonable indications for IM (Table 3). A recent systematic review and meta-analysis of complementary treatments for endometriosis reported that significant pain reduction is obtained with acupuncture in comparison with sham acupuncture [43]. Other complementary interventions studied included exercise, electrotherapy, and yoga. All of these were inconclusive in relation to benefit, but demonstrated a positive trend in the treatment of endometriosis symptoms [43].

The present study is not without limitations. The self-reported nature of the survey means that the data are at risk of responder and recall bias; there is an inherent bias when distributing a survey regarding IM because those who care for the matter tend to respond more than those who do not. Furthermore, since invitations were sent out by email and could have been forwarded to other physicians, a response rate cannot be calculated. It is also unclear whether responders were representative for the population of German gynecologists and onco-gynecologists. Another limitation is the missing answers in some questions. Moreover, most participants were from larger cities, which might also reduce generalizability; however, hospitals and private practices in Germany tend to be located in bigger cities rather than in smaller ones. Last but not least, most of the initiators of this survey work in the field of gynecology, and some work in the field of IM, and thus were not free of inherent preconceptions about the topic of this survey. This most likely did not lead to substantial bias, since data were assessed and analyzed quantitatively rather than qualitatively.

Despite these limitations, it is clear that physicians need to obtain information about the field of IM to provide accurate advice to patients and optimize their care.

Importantly, the representative national sample included in the study means that the findings may be generalized, and as such this study has potential value for German policy-makers, researchers, and health professionals.

## Conclusion

There is a high level of interest in IM among both office-based and hospital-based gynecologists. The availability of evidence-based training in IM is growing, and IM therapy approaches are being increasingly implemented in clinical routine work all over Germany. As IM therapies may involve potential hazards, the provision of qualified IM counseling integrated into conventional medicine may be a helpful tool for keeping in touch with patients who might otherwise withdraw from the physicians’ sphere of influence. Efforts should focus on extending evidence-based knowledge and integrating it into official medical guidelines. Physicians have an ethical obligation to optimize the use of resources and implement newly acquired evidence by incorporating evidence-based IM into conventional medicine and counseling patients proactively about evidence-based practices.

## Electronic supplementary material

Below is the link to the electronic supplementary material.

Supplementary file1 (DOCX 76 KB)

Supplementary file2 (DOCX 32 KB)

Supplementary file3 (DOCX 17 KB)

Supplementary file4 (DOCX 86 KB)

## Data Availability

Detailed data are available on request by email from Dr. Donata Grimm (d.grimm@uke.de and donatakatharina.grimm@uksh.de) and Dr. Carolin Hack (carolin.hack@uk-erlangen.de).
