# Supplementary material for: Gynecologists’ attitudes toward and use of complementary and integrative medicine approaches: results of a national survey in Germany
Source: Arch Gynecol Obstet. 2020 Nov 17;303(4):967–80. doi: 10.1007/s00404-020-05869-9 (PMC7985114; doi:10.1007/s00404-020-05869-9)
Supplement: Supplementary file 1 — Supplementary file1 (DOCX 76 KB) [file 404_2020_5869_MOESM1_ESM.docx]

**Supplementary digital file 1**

**Part 1: Selection of participants**

**Do you offer complementary medical treatment methods in your clinic or practice?**

- Yes
- No
- Not specified

**If NO, for what reasons? (multiple answers possible!)**

- I have no interest
- I am interested, but have too little knowledge or no special education or training
- I lack scientific evidence of complementary treatment methods
- I lack guidelines/recommendations for action regarding evidence-based methods
- I believe that complementary medicine does not bring any additional benefit
- I believe complementary medicine does more harm than good
- Other reasons: _____________________________________________________

**Are you planning to introduce complementary medicine procedures?**

- Yes
- No
- Not specified

**Part 2: Do you offer complementary medical treatment methods in your practice or surgery?**

1. **Are complementary medicine therapy procedures already routinely implemented in your therapy concept or are they an integral part of your therapy concept?**

- Yes, since ____ years
- No, not yet routinely
- No information

1. **When do you inform patients about complementary medicine treatment methods?**

- I always inform patients about additional complementary treatment options for the corresponding indication.
- I only inform patients with the appropriate indication if they themselves approach me about complementary treatment options/alternatives.
- I only inform patients with corresponding indications if conventional medical methods are not sufficient or have failed.
- No information

1. **Who advises patients on complementary medicine therapy methods?**

| Physician  Breast care nurse  Study nurse  Physiotherapist  Nutritionist / dietary assistant  Mind-body medical therapists  References to cooperation partners (e.g., alternative practitioner, other clinic, etc.)  Others: ________________________ | - Yes - Yes - Yes - Yes - Yes - Yes - Yes - Yes | - No - No - No - No - No - No - No - No | - Not specified - Not specified - Not specified - Not specified - Not specified - Not specified - Not specified - Not specified |
| --- | --- | --- | --- |

1. **What additional qualifications or areas of expertise regarding complementary therapy methods does the counselor have?**

| **Qualification training offered by state medical associations:**  Nutritional medicine  Acupuncture  Manual medicine/chirotherapy  Homeopathy  Naturopathy  **Other further education:**  Phytotherapy  Anthroposophic medicine  Neural therapy  TCM (traditional Chinese medicine)  Others:________________________________ | - Yes - Yes - Yes - Yes - Yes - Yes - Yes - Yes - Yes - Yes | - No - No - No - No - No - No - No - No - No - No | - Not specified - Not specified - Not specified - Not specified - Not specified - Not specified - Not specified - Not specified - Not specified - Not specified |
| --- | --- | --- | --- |

1. **Would you inform your patients more often about complementary medicine therapy options if they were reimbursed?**

- Yes
- No
- Not specified

1. **Do the complementary medicine therapy procedures at your clinic/practice cover costs?**

- Yes
- No
- Not specified

1. **Which subjective expectations do your patients talk about regarding complementary medicine treatment? (multiple answers possible!)**

- Increase in quality of life
- Alleviation of pregnancy complaints
- Strengthening of the immune system
- Improved stress and disease management
- Intention to have holistic treatment
- Desire to become more involved and active
- Dissatisfaction with standard therapy methods
- Healing with self-help
- Promotion of cancer healing
- Alleviation of side effects of the cancer therapy or the disease
- Prolongation of life (for cancer patients)
- Not specified

1. **Do you use complementary medical treatment methods in the field of obstetrics?**

- No, I do not use complementary medicine in the field of obstetrics (proceed directly to question 9)
- Yes, when there are: (multiple answers possible!)
  - Complaints during pregnancy
  - Complaints in the puerperium
  - Complaints during birth
  1. **Which complementary medical treatment methods do you offer or recommend to your patients in this area? (multiple answers possible!)**

| **Medical systems**  Therapies according to KNEIPP  Anthroposophic medicine  Classical homeopathy  TCM (including acupuncture/acupressure)   - Ayurveda   **Biological therapies**   - Mistletoe therapy - Phytotherapy - Food supplements (vitamins, minerals, trace elements, amino acids, fatty acids) - Cancer diets   **Further methods**   - Hyperthermia - Autologous blood - Aromatherapy - Wraps/rests - Others: | **Manipulative and body therapies**   - Osteopathy/chiropractic/ cranio-sacral therapy - Manual therapies (massage/lymphotherapy) - Neural therapy - **Sport/exercise therapy**   **Mind-body interventions**   - Autogenic training - Relaxation methods, e.g., progressive muscle relaxation - Biofeedback - Meditation - Hypnosis - Creative therapy (art/music) - Qigong, Tai Chi - Reiki - Yoga |
| --- | --- |

1. **Do you use complementary medical treatment methods in the field of general gynecology (non-gynecological oncology & obstetrics)?**

- No, I do not use complementary medicine in these areas (proceed directly to question 10)
- Yes, with: (multiple answers possible!)
  - Incontinence
  - Sterility
  - Climacteric complaints
  - Premenstrual syndrome
  - Polycystic ovarian syndrome
  - Hormonal dysregulation
  - Genital infections
  - Urinary tract infections
  - Endometriosis
  1. **Which complementary medical treatment methods do you offer or recommend to your patients in this area? (multiple answers possible!)**

| **Medical systems**  Therapies according to KNEIPP  Anthroposophic medicine  Classical homeopathy  TCM (including acupuncture/acupressure)   - Ayurveda   **Biological therapies**   - Mistletoe therapy - Phytotherapy - Food supplements (vitamins, minerals, trace elements, amino acids, fatty acids) - Cancer diets   **Further methods**   - Hyperthermia - Autologous blood - Aromatherapy - Wraps/rests - Others: | **Manipulative and body therapies**   - Osteopathy/chiropractic/ cranio-sacral therapy - Manual therapies (massage/lymphotherapy) - Neural therapy - **Sport/exercise therapy**   **Mind-body interventions**   - Autogenic training - Relaxation methods, e.g., progressive muscle relaxation - Biofeedback - Meditation - Hypnosis - Creative therapy (art/music) - Qigong, Tai Chi - Reiki - Yoga |
| --- | --- |

1. **Do you use complementary medical treatment methods in the field of gynecological oncology?**

- No, I do not use complementary medicine in the field of gynecological oncology (proceed directly to question 13)
- Yes, with: (multiple answers possible!)

| - **Malignancies**   - Breast cancer   - Ovarian cancer   - Fallopian tube/   peritoneal cancer   - - Cervical cancer   - Endometrial cancer   - Vulvar/vaginal cancer | **Side effects of cancer therapy**   - Nausea and vomiting - Loss of appetite - Abdominal complaints (constipation/diarrhea/pain) - (Tumor) pain - Polyneuropathy - Joint complaints - Mucositis - Skin changes (e.g., dermatitis under radiotherapy) - Hand-foot syndrome - Climacteric complaints - Fatigue/exhaustion - Insomnia - Psychological complaints, e.g., anxiety, depression - Problems with concentration/memory |
| --- | --- |

- 1. **Which complementary medical treatment methods do you offer or recommend to your patients in this area? (multiple answers possible!)**

| **Medical systems**  Therapies according to KNEIPP  Anthroposophic medicine  Classical homeopathy  TCM (including acupuncture/acupressure)   - Ayurveda   **Biological therapies**   - Mistletoe therapy - Phytotherapy - Food supplements (vitamins, minerals, trace elements, amino acids, fatty acids) - Cancer diets   **Further methods**   - Hyperthermia - Autologous blood - Aromatherapy - Wraps/rests - Others: | **Manipulative and body therapies**   - Osteopathy/chiropractic/ cranio-sacral therapy - Manual therapies (massage/lymphotherapy) - Neural therapy - **Sport/exercise therapy**   **Mind-body interventions**   - Autogenic training - Relaxation methods, e.g., progressive muscle relaxation - Biofeedback - Meditation - Hypnosis - Creative therapy (art/music) - Qigong, Tai Chi - Reiki - Yoga |
| --- | --- |

1. **In which specific phases of cancer or anti-tumor therapy do you use complementary medical procedures in your clinic/practice (outpatient and/or stationary)? (multiple answers possible!)**

- Outpatient
- Stationary

| Patients after diagnosis (currently no therapy planned)  Patients undergoing chemotherapy  Patients undergoing radiotherapy  Patients undergoing hormone therapy  Patients undergoing antibody therapy  Patients in the pre-final phase  Patients in aftercare  Patients undergoing palliative therapy | - Yes - Yes - Yes - Yes - Yes - Yes - Yes - Yes | - No - No - No - No - No - No - No - No | - Not specified - Not specified - Not specified - Not specified - Not specified - Not specified - Not specified - Not specified |
| --- | --- | --- | --- |

1. **If a patient asks for complementary therapies, what would you do?**

| Disease | Refer to the effectiveness  of conventional medicine | Apply methods from own practice | Referral to medical colleagues experienced in integrative medicine | Recommend an alternative practitioner | I don´t know |
| --- | --- | --- | --- | --- | --- |
| Breast  cancer |  |  |  |  |  |
| Ovarian  cancer |  |  |  |  |  |
| Cervical  cancer |  |  |  |  |  |
| Endometrial  cancer |  |  |  |  |  |
| Vulvar cancer |  |  |  |  |  |

1. **Do you work together with other professional groups in the field of complementary medicine? If so, please indicate the professional group(s) concerned.**

| - Yes - No - Not specified | - Specialist medical colleagues - Pharmacists - Midwives - Physiotherapists - Occupational therapists - Osteopaths - Nutritionists - Alternative practitioners___ - Others_____________________________ |
| --- | --- |

1. **Are you yourself a user of complementary medicine treatment methods?**

- Yes
- No
- Not specified

**Part 3: Demographic information about your person**

1. **Age:** _________
2. **Gender:** __female __male
3. **What is your highest academic title?**

- Doctor
- Private lecturer
- Professor
- No title

1. **What is your current training status regarding gynecology and obstetrics?**

- Resident
- Specialist in gynecology and obstetrics

1. **What additional qualifications or areas of expertise do you have?**

**(multiple answers possible!)**

- Nutritional medicine
- Homeopathy
- Naturopathy
- Acupuncture
- Emergency medicine
- Anthroposophic medicine
- Psycho-oncology, psychosomatics, psychology
- Others _________

1. **How many years have you been working as a gynecologist?**

Since ______ years

1. **Do you work as a registered gynecologist in a practice or are you employed in a clinic? (multiple answers possible!)**

- Established in a practice
- Clinic

1. **Are you working at a certified breast center or certified onco-gynecological center?**

| Certified breast center  Certified onco-gynecological center | - Yes - Yes | - No - No | - Not specified - Not specified |
| --- | --- | --- | --- |

1. **Where is your practice located?**

- Large city (> 100,000 inhabitants)
- Medium-sized City (20,000 - 100,000 inhabitants)
- Town (5,000 – 20,000 inhabitants)
- Rural region (< 5,000 inhabitants)

1. **In which federal state do you work?**

| - Baden-Wurttemberg - Bavaria - Berlin - Brandenburg - Bremen - Hamburg - Hesse - Mecklenburg-Western Pomerania | - Lower Saxony - North Rhine-Westphalia - Rhineland-Palatinate - Saarland - Saxony - Saxony-Anhalt - Schleswig-Holstein - Thuringia |
| --- | --- |

1. **How many patients do you treat in your clinic/practice per quarter?** ___________
2. **How many women with breast cancer come to your clinic/practice per year?**

**Please estimate the number!**

Primary: __________

Metastasized: __________

Aftercare __________

1. **How many women with gynecological malignoma come to your clinic/practice per year? Please estimate the number!**

Primary: __________

Metastasized: __________

Aftercare __________

1. **Which patients do you care for (in terms of health insurance)?**

- Patients covered by statutory health insurance, and private patients
- Only private patients
