# Supplementary material for: Gynecologists’ attitudes toward and use of complementary and integrative medicine approaches: results of a national survey in Germany
Source: Arch Gynecol Obstet. 2020 Nov 17;303(4):967–80. doi: 10.1007/s00404-020-05869-9 (PMC7985114; doi:10.1007/s00404-020-05869-9)
Supplement: Supplementary file 2 — Supplementary file2 (DOCX 32 KB) [file 404_2020_5869_MOESM2_ESM.docx]

**Supplementary digital file 2** Explanation of terms with regard to the topic of integrative and complementary medicine

| **Terms** | **Definition and explanation** |
| --- | --- |
| Alternative medicine | Alternative medicine refers to non-mainstream practices that are generally not considered standard medical approaches and are used instead of conventional medicine [1]. |
| Anthroposophic medicine | Anthroposophic medicine is a medical system based on a specific organismic concept and uses drugs derived from herbal, mineral, and animal sources, eurythmy (movement therapy), art therapy, rhythmical massage, and lifestyle recommendations. While not a recognized qualification for physicians, anthroposophic medicine is commonly used by German physicians [2]. |
| Ayurvedic Medicine | The ancient Indian medical system, also known as Ayurveda, is based on ancient writings that rely on a “natural” and holistic approach to physical and mental health. Ayurvedic medicine is one of the world’s oldest medical systems and remains one of India’s traditional health care systems. Ayurvedic treatment combines products (mainly derived from plants, but may also include animal, metal, and mineral), diet, exercise, and lifestyle [3]. |
| Breast care nurse | As a relatively new kind of specialist, a breast care nurse (BCN) works in the multidisciplinary care of breast cancer patients at breast centers. In Germany, in accordance with European Society of Mastology (EUSOMA) guidelines, special programs train nurses as BCNs to improve the quality of care for such patients; these programs’ curricula meet the international standards of a BCN qualification [4]. |
| Complementary medicine | Complementary medicine (CAM) refers to healthcare practices that are often based on traditional knowledge, but usually not part of conventional medicine, although they are sometimes applied in conjunction with it [5,6]. |
| Diet assistant | Diet assistant is the protected designation for a medical profession. The formerly two-year and now three-year training takes place at state-approved schools or at private schools and is concluded with an examination. The definition of "dietician" (dietitian) was adopted by the International Congress of Dietetic Associations (ICDA) and has been adopted by the European Federation of the Associations of Dietitians (EFAD):  "A dietitian is a person with a qualification in nutrition and dietetics recognized by national authorities. The dietitian applies the science of nutrition to the feeding and education of groups of people and individuals in health and disease. Dietitian is the only health profession in the field of dietetics and nutrition that can be recognized in other European countries on the basis of EU Directive 2005/36/EC [7].” |
| Homeopathy | Homeopathy, also known as homeopathic medicine, is a medical system that was developed in Germany more than 200 years ago. It’s based on two unconventional theories: > “Like cures like”—the notion that a disease can be cured by a substance that produces similar symptoms in healthy people > “Law of minimum dose”—the notion that the *lower* the dose of the medication, the *greater* its effectiveness. Many homeopathic products are so diluted that no molecules of the original substance remain.  Homeopathic products come from plants (such as red onion, arnica [mountain herb], poison ivy, belladonna [deadly nightshade], and stinging nettle), minerals (such as white arsenic), or animals (such as crushed whole bees). Homeopathic products are often made as sugar pellets to be placed under the tongue; they may also be in other forms, such as ointments, gels, drops, creams, and tablets. Treatments are “individualized” or tailored to each person—it’s common for different people with the same condition to receive different treatments [8]. |
| Integrative medicine | According to the National Center for Complementary and Integrative Health (NCCIH), integrative medicine (IM) differs from CAM because it combines conventional and complementary treatments in a coordinated way [5]. |
| Medical systems | Medical systems are systems with a comprehensive theoretical basis and an established philosophical body of thought, which have a long tradition and have developed over long periods of time. Applied practices are based on a theoretical explanatory model. Examples of medical systems are anthroposophic medicine, traditional Chinese medicine (TCM), Ayurveda, traditional naturopathy and homeopathy [2]. |
| Mind-body therapist | A mind-body therapist works with the body to affect the mind and, for example, teaches yoga, progressive relaxation, meditation, and mindfulness. The “mind” is not synonymous with the brain, but consists of mental states such as thoughts, emotions, beliefs, attitudes, and images, all of which can have an effect on a person’s physical well-being [9,10]. |
| Naturopathy | Naturopathy is used internationally for different and often eclectic treatment approaches, although it mainly encompasses herbal medicine (also called phytotherapy), hydrotherapy, and mind-body medicine counseling in Germany [2]. |
| Nutritionist | Nutritional medicine is an interdisciplinary medical discipline that uses current scientific knowledge about the physiology and pathophysiology of human nutrition to prevent, cure and alleviate disease. Additional training in nutritional medicine includes the recognition, treatment and prevention of nutrition-dependent diseases caused by congenital or acquired metabolic disorders [11]. |
| Phytotherapy | Phytotherapy is the use of plant-derived medications in the treatment and prevention of disease. Phytotherapy is a science-based medical practice and thus is distinguished from other, more traditional approaches, such as medical herbalism [12]. |
| Sports scientist | A sports scientist in Germany has an academic degree in the study of how the healthy human body works during exercise, and how sport and physical activity promote health. Sports science incorporates aspects of physiology (exercise), psychology, anatomy, biomechanics, biochemistry, and biokinetics. The scientific study of sport allows researchers to develop a better understanding of how the human body reacts to exercise, varying environments, and many other stimuli [13]. |
| Study nurse | A study nurse or study assistant is a person who supervises clinical studies in study centers of hospitals, medical practices or the pharmaceutical industry. The study nurse is jointly responsible for the conduct of the study in accordance with the study protocol, acting as the interface between patients, investigators, monitors of the contract research organization (CRO), and the pharmaceutical industry as well as university hospitals or study groups as the sponsor of the study. The activities of the study nurse are based on good clinical practice (GCP) and the German Medicines Act (AMG).  Study nurse is not a protected occupational title; there is no uniform regulation for further education. A basic medical education such as that for a medical assistant (called physician assistant until 2006) or nurse, as well as regular further education are helpful; English and computer skills are required. Since the job description for the study nurse is not clearly defined, the field of activity can vary greatly, depending on the employer and the area of responsibility [14]. |

**References**

1. National Cancer Institute (2019) Complementary and alternative medicine. <https://www.cancer.gov/about-cancer/treatment/cam>. Accessed Oct 13, 2020 2020

2. Hack CC, Hüttner NBM, Paepke D, Voiss P, dobos G, Kümmel S, Münstedt K, Kiechle M, Fasching PA, Beckmann MW (2013) Integrative Medizin in der Gynäkologischen Onkologie – Möglichkeiten und Grenzen Teil 1. Geburtsh Frauenheilk 73:R65-R80

3. National Center for Complementary and Integrative Health (2019) Ayurvedic medicine: in depth. <https://www.nccih.nih.gov/health/ayurvedic-medicine-in-depth>. Accessed Oct 19, 2020 2020

4. Voigt B, Grimm A, Lossack M, Klose P, Schneider A, Richter-Ehrenstein C (2011) The breast care nurse: the care specialist in breast centres. International nursing review 58 (4):450-453. doi:10.1111/j.1466-7657.2011.00893.x

5. National Center for Complementary and Integrative Health (2018) Complementary alternative, or integrative health: what’s in a name? <https://www.nccih.nih.gov/health/complementary-alternative-or-integrative-health-whats-in-a-name>. Accessed Oct 13, 2020 2019

6. World Health Organization (2020) Traditional, complementary and integrative medicine. <https://www.who.int/health-topics/traditional-complementary-and-integrative-medicine#tab=tab_1>. Accessed Oct 13, 2020

7. European Federation of the Associations of Dietitians (2016) Definition of a dietitian. <http://www.efad.org/en-us/about-efad/definition-of-a-dietitian/#:~:text=A%20dietitian%20is%20a%20person,individuals%20in%20health%20and%20disease>. Accessed Oct 13, 2020 2020

8. Health NCfCaI (2018) Homeopathy. <https://www.nccih.nih.gov/health/homeopathy>. Accessed Oct 19, 2020 2020

9. Integrative Medical Institute (2020) Mind-body therapies. <https://integrative-med.org/services/mind-body-therapies/>. Accessed Oct 13, 2020 2020

10. Dobos G, Altner N, Lange S, Musial F, Langhorst J, Michalsen A, Paul A (2006) Mind-body medicine as a part of German integrative medicine. Bundesgesundheitsblatt, Gesundheitsforschung, Gesundheitsschutz 49 (8):723-728. doi:10.1007/s00103-006-0001-0

11. Kris-Etherton PM, Akabas SR, Bales CW, Bistrian B, Braun L, Edwards MS, Laur C, Lenders CM, Levy MD, Palmer CA, Pratt CA, Ray S, Rock CL, Saltzman E, Seidner DL, Van Horn L (2014) The need to advance nutrition education in the training of health care professionals and recommended research to evaluate implementation and effectiveness. The American journal of clinical nutrition 99 (5 Suppl):1153s-1166s. doi:10.3945/ajcn.113.073502

12. Britannica (2017) Phytotherapy. <https://www.britannica.com/science/phytotherapy>. Accessed Oct 13, 2020 2020

13. Medical School Hamburg (2020) Studium Sportwissenschaft: Leistungsdiagnostik und Trainingssteuerung (Master of Science). <https://www.medicalschool-hamburg.de/studiengaenge/fakultaet-gesundheitswissenschaften-fachhochschule/masterstudiengaenge/sportwissenschaft-leistungsdiagnostik-und-trainingssteuerung/?gclid=EAIaIQobChMIr_Pbt4mx7AIVh63tCh2fwAbwEAAYASAAEgLB6vD_BwE>. Accessed Oct 13, 2020 2020

14. Sudhop T (2000) Study Nurse – Notwendigkeit einer standardisierten und qualifizierten Ausbildung. Deutsche Zeitschrift für Klinische Forschung 2 (4)
