# Supplementary material for: Gynecologists’ attitudes toward and use of complementary and integrative medicine approaches: results of a national survey in Germany
Source: Arch Gynecol Obstet. 2020 Nov 17;303(4):967–80. doi: 10.1007/s00404-020-05869-9 (PMC7985114; doi:10.1007/s00404-020-05869-9)
Supplement: Supplementary file 3 — Supplementary file3 (DOCX 17 KB) [file 404_2020_5869_MOESM3_ESM.docx]

**Supplementary digital file 3** Integrative Therapies During and After Breast Cancer Treatment

In 2017, the Society for Integrative Oncology (SIO) produced an evidence-based guideline on the use of integrative therapies during and after breast cancer treatment. In 2018, the American Society of Clinical Oncology (ASCO) endorsed the guideline with a few added discussion points. In the following, we list the key recommendations by SIO as endorsed by ASCO [1].

**Acute Radiation Skin Reaction**

- Aloe vera and hyaluronic acid cream should not be recommended for improving acute radiation skin reaction. (Grade D)

**Anxiety and Stress Reduction**

- Meditation is recommended for reducing anxiety. (Grade A)
- Music therapy is recommended for reducing anxiety. (Grade B)
- Stress management is recommended for reducing anxiety during treatment, but longer group programs are likely better than self-administered home programs or shorter programs. (Grade B)
- Yoga is recommended for reducing anxiety. (Grade B)
- Acupuncture, massage, and relaxation can be considered for reducing anxiety. (Grade C)

**Chemotherapy-Induced Nausea and Vomiting**

- Acupressure can be considered as an addition to antiemetic drugs to control nausea and vomiting during chemotherapy. (SIO: Grade B; ASCO: Grade C)
- Electroacupuncture can be considered as an addition to antiemetic drugs to control vomiting during chemotherapy. (SIO: Grade B; ASCO: Grade C)
- Ginger and relaxation can be considered as additions to antiemetic drugs to control nausea and vomiting during chemotherapy. (Grade C)
- Glutamine should not be recommended for improving nausea and vomiting during chemotherapy. (Grade D)

**Depression and Mood Disturbance**

- Meditation, particularly mindfulness-based stress reduction, is recommended for treating mood disturbance and depressive symptoms. (Grade A)
- Relaxation is recommended for improving mood disturbance and depressive symptoms. (Grade A)
- Yoga is recommended for improving mood disturbance and depressive symptoms. (Grade B)
- Massage is recommended for improving mood disturbance. (Grade B)
- Music therapy is recommended for improving mood disturbance. (Grade B)
- Acupuncture, healing touch, and stress management can be considered for improving mood disturbance and depressive symptoms. (Grade C)

**Fatigue**

- Hypnosis and ginseng can be considered for improving fatigue during treatment. (Grade C)
- Acupuncture and yoga can be considered for improving post-treatment fatigue. (Grade C)
- Acetyl-l-carnitine and guarana should not be recommended for improving fatigue during treatment. (Grade D)

**Lymphedema**

- Low-level laser therapy, manual lymphatic drainage, and compression bandaging can be considered for improving lymphedema. (Grade C)

**Neuropathy**

- Acetyl-l-carnitine is not recommended for the prevention of chemotherapy-induced peripheral neuropathy in patients with breast cancer due to potential harm. (Grade H)

**Pain**

- Acupuncture, healing touch, hypnosis, and music therapy can be considered for the management of pain. (Grade C)

**Quality of Life**

- Meditation is recommended for improving quality of life. (Grade A)
- Yoga is recommended for improving quality of life. (Grade B)
- Acupuncture, mistletoe, qigong, reflexology, and stress management can be considered for improving quality of life. (Grade C)

**Sleep Disturbance**

- Gentle yoga can be considered for improving sleep. (Grade C)

**Vasomotor/Hot Flashes**

- Acupuncture can be considered for improving hot flashes. (Grade C)
- Soy is not recommended for hot flashes in patients with breast cancer due to lack of effect. (Grade D)

**Reference:**

1. Lyman GH, Greenlee H, Bohlke K, et al. Integrative Therapies During and After Breast Cancer Treatment: ASCO Endorsement of the SIO Clinical Practice Guideline. J Clin Oncol. 2018;36(25):2647-2655.
