# Supplementary material for: Gynecologists’ attitudes toward and use of complementary and integrative medicine approaches: results of a national survey in Germany
Source: Arch Gynecol Obstet. 2020 Nov 17;303(4):967–80. doi: 10.1007/s00404-020-05869-9 (PMC7985114; doi:10.1007/s00404-020-05869-9)
Supplement: Supplementary file 4 — Supplementary file4 (DOCX 86 KB) [file 404_2020_5869_MOESM4_ESM.docx]

**Supplementary digital file 4**

**Fig. 3** Provision of integrative medicine (IM) treatment methods by onco-gynecologists relative to different phases of treatment (n = 113) for patients with cancer diseases. Multiple responses were allowed.

Only subjects responding “Yes” to the question “Do you use complementary medical treatment methods in the field of gynecological oncology?” were included.
